# In vivo characterization of the BLV miRNA cluster

**DOI:** 10.1186/1742-4690-12-S1-P15

**Published:** 2015-08-28

**Authors:** Anthony Rodari, Benoît Van Driessche, Nadège Delacourt, Caroline Vanhulle, Arsène Burny, Anne Van den Broeke, Olivier Rohr, Carine Van Lint

**Affiliations:** 1 Service of Molecular Virology, University of Brusells (ULB), Gosselies, Belgium; 2 Laboratory of Experimental Hematology, Institut Jules Bordet, University of Brusells (ULB), Brusells, Belgium; 3 Unit of Animal Genomics, GIGA, University of Liège (ULg), Liège, Belgium; 4 Institut Universitaire de Technologie (IUT) Louis Pasteur de Schiltigheim, Université of Strasbourg, Schiltigheim, France

## 

Bovine leukemia virus (BLV), a B-lymphotropic oncogenic retrovirus sharing common biological and structural features with the human T-cell leukemia virus I and II (HTLV-I and II), is the etiologic agent of enzootic bovine leucosis. One major feature of the BLV infection is the absence of viremia. It is widely accepted that BLV latency, due to the RNA polymerase II 5’-LTR-driven transcriptional and epigenetic repression is a viral strategy to escape from the host immune system and allow tumor development. Recently, it has been demonstrated by deep sequencing and bioinformatics analysis that the BLV genome encodes a cluster of micro-RNAs which is predicted to be transcribed by RNA polymerase III suggesting that the silencing dogma in BLV transcriptional regulation is only partially correct. Here, we demonstrated by chromatin immunoprecipitation assays that BLV miRNAs were indeed transcribed in vivo by RNA polymerase III through a type II RNAPIII promoter similar to the one directing tRNAs transcription. We also showed that an activating epigenetic environment surrounds the miRNAs cluster. Overall, our results provide new insights into a better understanding of the molecular mechanisms regulating gene expression of oncogenic retroviruses.

